# Atomic threshold-switching enabled MoS_2_ transistors towards ultralow-power electronics

**DOI:** 10.1038/s41467-020-20051-0

**Published:** 2020-12-04

**Authors:** Qilin Hua, Guoyun Gao, Chunsheng Jiang, Jinran Yu, Junlu Sun, Taiping Zhang, Bin Gao, Weijun Cheng, Renrong Liang, He Qian, Weiguo Hu, Qijun Sun, Zhong Lin Wang, Huaqiang Wu

**Affiliations:** 1 Institute of Microelectronics, Beijing Innovation Center for Future Chips (ICFC), Tsinghua University, 100084 Beijing, China; 2 CAS Center for Excellence in Nanoscience, Beijing Key Laboratory of Micro-nano Energy and Sensor, Beijing Institute of Nanoenergy and Nanosystems, Chinese Academy of Sciences, 101400 Beijing, China; 3 School of Nanoscience and Technology, University of Chinese Academy of Sciences, 100049 Beijing, China; 4 Department of Electrical Engineering, Tsinghua University, 100084 Beijing, China; 5 School of Materials Science and Engineering, Georgia Institute of Technology, Atlanta, GA 30332-0245 USA

**Keywords:** Electrical and electronic engineering, Electronic devices, Electronic devices

## Abstract

Power dissipation is a fundamental issue for future chip-based electronics. As promising channel materials, two-dimensional semiconductors show excellent capabilities of scaling dimensions and reducing off-state currents. However, field-effect transistors based on two-dimensional materials are still confronted with the fundamental thermionic limitation of the subthreshold swing of 60 mV decade^−1^ at room temperature. Here, we present an atomic threshold-switching field-effect transistor constructed by integrating a metal filamentary threshold switch with a two-dimensional MoS_2_ channel, and obtain abrupt steepness in the turn-on characteristics and 4.5 mV decade^−1^ subthreshold swing (over five decades). This is achieved by using the negative differential resistance effect from the threshold switch to induce an internal voltage amplification across the MoS_2_ channel. Notably, in such devices, the simultaneous achievement of efficient electrostatics, very small sub-thermionic subthreshold swings, and ultralow leakage currents, would be highly desirable for next-generation energy-efficient integrated circuits and ultralow-power applications.

## Abstract (web summary)

Here, the authors demonstrate an atomic threshold-switching field-effect transistor constructed by integrating a metal filamentary switch with a two-dimensional MoS_2_ channel, and obtain abrupt steepness in the turn-on characteristics and 4.5 mV/dec subthreshold swing over five decades.

## Introduction

Scaling-down of complementary metal–oxide–semiconductor (CMOS) field-effect transistors (FETs) is a mainstream approach for reducing power dissipation in the rapidly developing field of information technology^1–4^. However, CMOS technology still faces a great challenge of feature size <5 nm, due to the degradation of the off-state leakage current induced by short-channel effects (i.e., direct source–drain punch through, and a loss of gate electrostatic control)^3–8^. An efficient way to minimize power consumption is to achieve a steep subthreshold swing (SS) with a fast-switching rate at a reduced supply voltage^7,9^. The emerging two-dimensional (2D) transition metal dichalcogenides^10–12^, e.g., atomically thin molybdenum disulfide (MoS_2_)^5,8,13^, are promising channel materials for future electronic chips with scaling dimensions and ultralow off-state currents, due to the high electron effective mass, low dielectric constant, and large bandgap^10–13^. Furthermore, the atomic-scale thickness and smoothness of MoS_2_ also significantly improve the electrostatic gate control capability according to its characteristic scaling length and can efficiently lower the supply voltage^8,10^. However, the electrons in the source usually represent a thermal Boltzmann distribution with a potential barrier, which restricts the gate modulation capacity to 60 mV decade^−1^, as determined by the thermal voltage (*kT*/*q*)^6,7^. Hence, to break the “Boltzmann tyranny”, enabling atomic-scale FETs with steep subthreshold behavior and still maintaining high on/off current ratio, is critical for the development of ultralow-power electronics.

Several strategies have been proposed to obtain an SS of sub-60 mV decade^−1^, such as band-to-band tunneling^8^, impact ionization^14^, nanoelectromechanical switching^15^, Dirac-source^16^, negative capacitance (NC)^17–20^, and negative differential resistance (NDR)^21,22^. The demonstrated threshold-switching behavior acting as an internal amplifier offers a shortcut to conquer the Boltzmann limit and triggers the FET to switch with a sub-*kT*/*q* slope. In particular, the NDR effect in threshold-switching FET is highly predictable and quantifiable for constructing steeply switchable electronic devices with high performance^9,21^. When compared to a common insulator–metal–transition device (e.g., VO_2_)^21,23^, a new type of metal filamentary threshold switch (TS), which generally consists of Ag (or Cu) as an active electrode or dopant in a solid electrolyte, has been demonstrated a lower leakage current and much steeper switching characteristics^24–27^, and can contribute to suppressing the off-state leakage current of conventional FETs with an abrupt SS^22,28^. The seamless device architecture based on a 2D FET and TS may realize the simultaneous achievement of efficient electrostatic control, small sub-thermionic SS, and low leakage current, leading to efficiently minimizing power dissipation.

Here, we present an atomic threshold-switching MoS_2_ FET (ATS-FET) with sharp on/off switching properties and ultralow energy consumption. The ATS-FET is endowed with an abrupt amplification capacity via integrating an abrupt variable-resistance Ag atomic TS with an atomically thin MoS_2_ channel. The channel thickness and metal filament at the atomic scale are critical to reducing the supply voltage. The common HfO_2_ insulator functions as the dielectric layer for the FET and the electrolyte for the TS, which is promising for future monolithic integration of the ATS-FET configurations with facile fabrication processes. The NDR effect according to the abrupt resistance transition of the TS induces an internal voltage amplification across the MoS_2_ channel, which enables the MoS_2_ FET to significantly overcome the fundamental thermionic limitation. According to the atomic thickness of the MoS_2_ channel and the Ag atomic conductive filament (Ag filament), the superior electrical performances, such as on/off current ratio >10^6^, ultralow cutoff current at 1 × 10^−13^ A μm^−1^, an average SS (SS_average_) of sub-5 mV decade^−1^ (median), and very small hysteresis, are achieved in the ATS-FET. We systematically investigate the influence of the NDR effect on the internal amplification phenomena and evaluate the improved electrical performance of the ATS-FET. The proposed ATS-FET has great potential to be extended to scalable and monolithic steep-slope transistor arrays and is of great significance in energy-efficient and high-performance electronic switches with ultralow-power dissipation.

## Results

### ATS-FET design

**Fig. 1:**
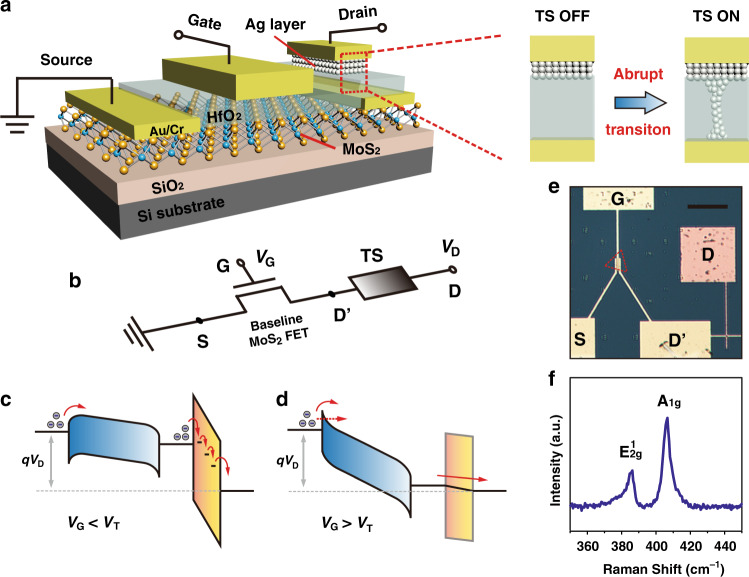
Ultralow-power steep-slope 2D MoS_2_ field-effect transistor with Ag atomic threshold-switching (ATS-FET). **a** Schematic illustration of the ATS-FET, consisting of a MoS_2_ FET and an Ag/HfO_2_ based TS. The enhanced performance of the ATS-FET is essentially attributed to the abrupt switching transition of the TS. **b** The equivalent circuit schematic of the ATS-FET, which can be considered as a baseline MoS_2_ FET in series with a TS device. *V*_D_ is the drain-to-source voltage across the TS and the MoS_2_ FET; *V*_G_ is the gate-to-source voltage across the MoS_2_ FET. **c**, **d** Schematic band diagrams of the ATS-FET under thermal equilibrium with *V*_G_ bias, including **c** *V*_G_ < *V*_T_ and **d** *V*_G_ > *V*_T_. Electrons pass through the contact barriers via thermionic emission or tunneling; and across the electrolyte barrier via hopping. **e** Optical image of the ATS-FET device structure, exhibiting that the TS stack (cross-point area: 2 × 2 μm^2^) in series with the channel of the MoS_2_ FET. The red-dashed triangle indicates the MoS_2_ channel material. Scale bar: 50 μm. **f** Raman spectrum of the CVD synthesized MoS_2_ nanoflake. The peaks of in-plane *E*_2g_ and out-of-plane *A*_1g_ vibrational modes are at 386.2 and 406.6 cm^−1^, respectively.

Figure 1a shows a schematic illustration of the ATS-FET by integrating an Ag atomic TS in series with a MoS_2_ FET. The FET shares the same HfO_2_ dielectric with the TS (acting as the electrolyte in the TS). Triangle-shaped MoS_2_ nanoflakes are first synthesized on a SiO_2_/Si substrate by chemical vapor deposition (CVD). The source/drain electrodes (Cr/Au) top-contacted with the MoS_2_ are prepared via standard e-beam lithography (EBL), thermal evaporation, and lift-off process. The common HfO_2_ thin film is deposited with atomic layer deposition (ALD), functioning as both the dielectric layer of the FET and the electrolyte of the TS. The Ag atomic layer is patterned on HfO_2_ by a standard liftoff process. Top gate and drain contacts are defined at the desired position via EBL and metallization. The structural design of the common HfO_2_ layer simplifies the fabrication process, while ensuring a high-*κ* dielectric performance for FET operation and good electrochemical kinetics for threshold switching. The fast formation and spontaneous rupture of the atomic Ag filament in the TS will lead to abrupt on/off switching in the ATS-FET with an ultralow SS (Fig. 1a).

Figure 1b depicts the equivalent circuit diagram of the ATS-FET, which can be considered as a baseline MoS_2_ FET in series with a TS device. The supply voltage (or drain-to-source voltage, *V*_D_) drives the TS and the MoS_2_ channel, while the gate-to-source voltage (*V*_G_) controls the switching characteristics of the ATS-FET. The supply voltage variably distributes between the TS and FET, corresponding to a voltage drop of *V*_D_ for the whole device and *V*_D′_ for the MoS_2_ FET, respectively. Based on the series TS configuration, *V*_G_ can tune the Fermi level of the MoS_2_ channel and lead to efficient control of the channel resistance, which dominantly determines the voltage drop between the FET and TS in the series configuration. The band diagrams of the ATS-FET for a typical *V*_G_ (compared with the threshold voltage, *V*_T_) are illustrated in Fig. 1c, d. The HfO_2_ electrolyte layer in the series TS can be considered as a variable barrier for electron transport according to the applied *V*_G_, which determines the voltage drop on the MoS_2_ channel and the TS component. For *V*_G_ < *V*_T_, the Fermi level of MoS_2_ is slightly shifted downward (the MoS_2_ channel resistance is maintained at a high level). The current across the TS is insufficient to trigger the bridging of the Ag filament. The HfO_2_ electrolyte layer acts as a large barrier to blocking the transport of electrons. When *V*_G_ > *V*_T_, the MoS_2_ Fermi level is effectively shifted downwards (the MoS_2_ channel resistance transits to a low level). The current across the TS could induce the Ag filament formation, leading to an abrupt current increase (the mechanism will be discussed below in detail). An optical image of the ATS-FET device is shown in Fig. 1e. The TS stack (Au/Ag/HfO_2_/Au) is connected in series to the FET, sharing its top electrode as the drain electrode of the ATS-FET. Figure 1f shows the Raman spectrum of a CVD synthesized MoS_2_ nanoflake excited with a 532 nm laser. The peaks of the in-plane *E*_2g_ and out-of-plane *A*_1g_ vibration modes at 386.2 and 406.6 cm^−1^, respectively, indicate that the as-grown MoS_2_ is a monolayer.

### ATS-FET versus the baseline FET

**Fig. 2:**
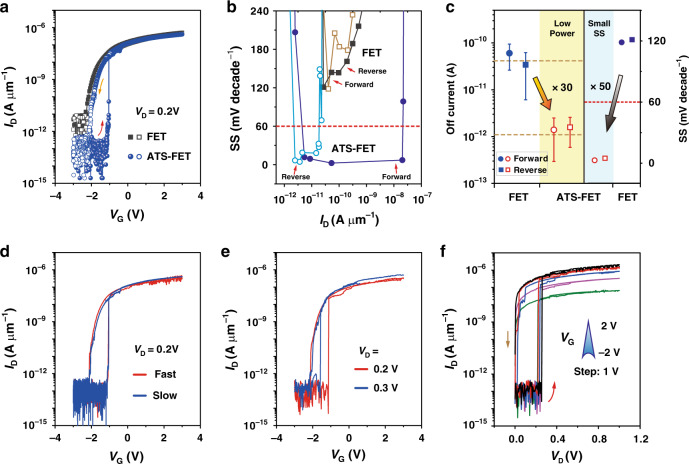
Device performances of the ATS-FET. **a** Transfer curves from the ATS-FET and FET with the same 2D MoS_2_ channel (channel width: 10 μm; channel length: 2 μm). Solid symbols indicate the forward sweep, while open symbols indicate the reverse sweep. **b** SS from the ATS-FET and FET both in forward and reverse sweeps. The FET operates well above the fundamental thermionic limitation, while the ATS-FET has a large range of the drain current where the minimal SS is 2.5 mV decade^−1^. **c** The off-state leakage current and SS can be simultaneously reduced in the ATS-FET, indicating the ultralow-power steep-slope phenomenon in the ATS-FET. **d** *I*_D_–*V*_G_ characteristics measured at room temperature and at *V*_D_ = 0.2 V at slow/fast gate voltage sweep speeds. The *V*_G_ steps were set to 3 and 30 mV, respectively. **e** Transfer characteristics (*I*_D_–*V*_G_) measured at room temperature and at *V*_D_ = 0.2 and 0.3 V. **f** Output characteristics (*I*_D_–*V*_D_) measured at room temperature at *V*_G_ from −2 to 2 V with a step of 1 V.

The transfer characteristics (*I*_D_–*V*_G_) of the ATS-FET and the baseline MoS_2_ FET are presented in Fig. 2a. As is clearly shown, when *V*_G_ sweeps from −3 to 3 V and back to −3 V, the baseline MoS_2_ FET exhibits an on/off current ratio of 10^5^ and a very small hysteresis (grey square curves). The minimal SS (SS_min_) in forward and reverse sweeps are characterized to be 118.3 and 120.9 mV decade^−1^, respectively, which are both above the thermionic limit of 60 mV decade^−1^ at room temperature (Fig. 2b). In contrast, the ATS-FET shows more superior electrical properties, including a higher on/off current ratio (5 × 10^6^), a lower off-state current (1 × 10^−13^ A μm^−1^), and a much steeper SS (<5 mV decade^−1^). It is remarkable that, in the ATS-FET, the series integration of a TS in the baseline MoS_2_ FET enables to produce an internal amplification to overcome the fundamental thermionic limitation of the Boltzmann distribution of electrons. More impressively, the exponentially increased transfer curves of the baseline MoS_2_ FET can transit to the nearly vertical transfer curves with much steeper slopes for the ATS-FET (Fig. 2a).

In the series combination, *V*_D_ is divided between the MoS_2_ channel and the TS corresponding to their individual resistances. The off-state (or leakage) current of ATS-FET is commonly determined by the ultrahigh resistance of the TS, leading to a further decrease of *I*_D_ to a lower level of 1 × 10^−13^ A μm^−1^. Initially, at a low *V*_G_, the *I*_D_ flowing through the MoS_2_ channel and TS is insufficient to induce the switching of TS. As *V*_G_ increases (i.e., forward sweep), the MoS_2_ channel resistance shows a gradual decrease until *I*_D_ approaches a critical threshold (*I*_th-TS_), which is capable to induce the switching on of TS (to low-resistance state, LRS). And consequently, the total resistance of the ATS-FET dramatically decreases and triggers an abrupt *I*_D_ increase. On the contrary, as *V*_G_ decreases (i.e., reverse sweep), the MoS_2_ channel resistance shows a gradual increase until *I*_D_ reduces to another critical threshold (*I*_hold-TS_), which could reversely induce the switching off of TS (back to high-resistance state, HRS) and causes a rapid drop of *I*_D_. Hence, hysteresis in the transfer curve can be found as a result of the difference in *V*_G_ corresponding to the two critical thresholds (Fig. 2a). Different from that of the baseline FET (clockwise), the hysteresis of the ATS-FET resembles an anticlockwise transition that is caused by the series integration of the TS.

From the subthreshold region of the ATS-FET (in Fig. 2a), the extracted SS_min_ in forward and reverse sweeps are 2.5 and 4.5 mV decade^−1^, respectively, as shown in Fig. 2b. Besides, the ATS-FET has a large range of *I*_D_ (over four decades) where the average SS (SS_average_) is 3.0 mV decade^−1^ in the forward sweep. It is considered that the NDR effect originating from the volatile threshold-switching behavior in the atomic Ag filament device induces an efficient internal voltage amplification across the atomically thick MoS_2_ channel, and contributes to inducing a record and significantly reduced SS, which is much smaller than the values previously reported for a tunnel FET (TFET) at 31.1 mV decade^−1^ (ref. ^8^), an NC-FET at 41.7 mV decade^−1^ (ref. ^19^), a Dirac-source CNT FET (DS-FET) at 35 mV decade^−1^ (ref. ^16^), and an ion liquid gating FET at 50 mV decade^−1^ (ref. ^29^). Moreover, the subthreshold characteristics of various types of the steep-slope MoS_2_ FETs are outlined in Supplementary Table 1. A record on SS_min_ of 0.3 mV decade^−1^ and SS_average_ of 1.3 mV decade^−1^ (over three decades) is also achieved in the ATS-FET at room temperature.

### Both reductions in off-state current and SS in the ATS-FET

Power dissipation is a fundamental issue for advanced CMOS technology, which faces two severe challenges: the increasing difficulty for the supply voltage scaling, and the rising leakage currents causing on/off current ratio degradation^6,7^. The energy efficiency of a logic operation can be estimated through the total switching energy (*E*_total_) consisting of dynamic (*E*_dynamic_) and static (*E*_static_) parts, defined as:

1 $$\begin{array}{*{20}{l}} {E_{{\mathrm{total}}}} \hfill & = \hfill & {E_{{\mathrm{dynamic}}} + E_{{\mathrm{static}}}} \hfill \\ {\,} \hfill & = \hfill & {\alpha C_{\mathrm{L}}V_{\mathrm{D}}^2 + I_{{\mathrm{off}}}V_{\mathrm{D}}\tau _{{\mathrm{delay}}}} \hfill \\ {\,} \hfill & \approx \hfill & {C_{\mathrm{L}}V_{\mathrm{D}}^2\left( {\alpha + \gamma 10^{\frac{{ - V_{\mathrm{D}}}}{{\mathrm{SS}}}}} \right)} \hfill \end{array},$$

where *C*_L_ is the switched capacitance, *τ*_delay_ is the delay time, *α* is the activity factor, and *γ* is a fitting parameter^7^. It can be inferred from the above equations that the steeper SS and a lower off-state current in FETs enable further scaling of the supply voltage and a corresponding reduction in total power dissipation. As shown in Fig. 2c (orange region), the integration of the TS with the baseline MoS_2_ FET helps to significantly suppress the off-state leakage currents by ~30 times, attributing to the ultrahigh resistance of the TS in the off-state (~1 TΩ). Besides, SS_min_ of the ATS-FET in forward and reverse sweeps have been demonstrated to greatly decrease to 2.5 and 4.5 mV decade^−1^, respectively. Both of them are far lower than the fundamental thermionic limitation and those of the baseline MoS_2_ FET (close to 50 times reduction, the cyan region of Fig. 2c).

### Electrical properties of the ATS-FET

To exclude the effects of the *V*_G_ sweeping rate on the ultralow SS, the transfer characteristics (*I*_D_–*V*_G_) of the ATS-FET are measured at slow and fast *V*_G_ sweep speeds of 3 and 30 mV s^−1^, respectively. As illustrated in Fig. 2d, identical steep-slope switching characteristics with high on/off current ratios over 10^6^ are observed at different *V*_G_ sweep speeds. The SS, *V*_T_, off-state current, and on/off ratio are all independent of the *V*_G_ sweeping rate. In addition, the transfer characteristics (*I*_D_–*V*_G_) of the ATS-FET at *V*_D_ = 0.2 and 0.3 V are shown in Fig. 2e. In the forward sweep, the SS_average_ at *V*_D_ = 0.2 and 0.3 V are characterized to be 4.5 mV decade^−1^ (over five decades) and 6.0 mV decade^−1^ (over four decades), respectively. Meanwhile, *V*_T_ of the ATS-FET shows a negative shift from −1.14 to −1.59 V, which is determined by the balances between the supply voltage and the relevant potential drops on the FET and TS during the *V*_G_ sweeping. At a higher *V*_D_, the TS favors a tendency for the turn on state; consequently, the abrupt resistance change is less efficient, and it is easier for the TS to be switched on at a lower *V*_G_. In the reverse sweep, the *I*_D_ of the ATS-FET decreases until it reaches the critical threshold *I*_hold-TS_ (<5 × 10^−11^ A μm^−1^), leading to the instantaneous switching off of the TS. And hence, the ATS-FET shows similar abrupt switching characteristics in the subthreshold region regardless of *V*_D_. The output characteristics (*I*_D_–*V*_D_) of the ATS-FET for different *V*_G_ are characterized, as shown in Fig. 2f. The channel current *I*_D_ increases from 6.7 × 10^−8^ to 2.1 × 10^−6^ A μm^−1^ as *V*_G_ increases from −2 to 2 V in the linear/saturation region, showing an increase in the channel conductance with increasing *V*_G_. Distinguishable on- and off-states are observed in different *V*_D_ regions (*V*_D_ < *V*_th-TS_ or *V*_D_ ≥ *V*_th-TS_, *V*_th-TS_ is the threshold voltage of the TS), as shown in Fig. 2f. As *V*_D_ increases, when *V*_D_ < *V*_th-TS_, the conductance of the ATS-FET is primarily determined by the TS, even though the MoS_2_ channel is in a LRS; when *V*_D_ ≥ *V*_th-TS_, the conductance of the ATS-FET is coordinated by the MoS_2_ channel. Similarly, as *V*_D_ decreases, the conductance of the ATS-FET shows a sudden reduction when *V*_D_ ≤ *V*_hold-TS_ (*V*_hold-TS_ is the hold voltage of the TS). Moreover, the output characteristics of another ATS-FET and its baseline FET at different *V*_G_ (from −2 to 2 V) in linear scale are also demonstrated in Supplementary Fig. 1. It is clearly observed that the abrupt switching on/off behavior of TS contributes to causing the steep-slope phenomenon of the 2D FET.

### Ag atomic threshold switching

**Fig. 3:**
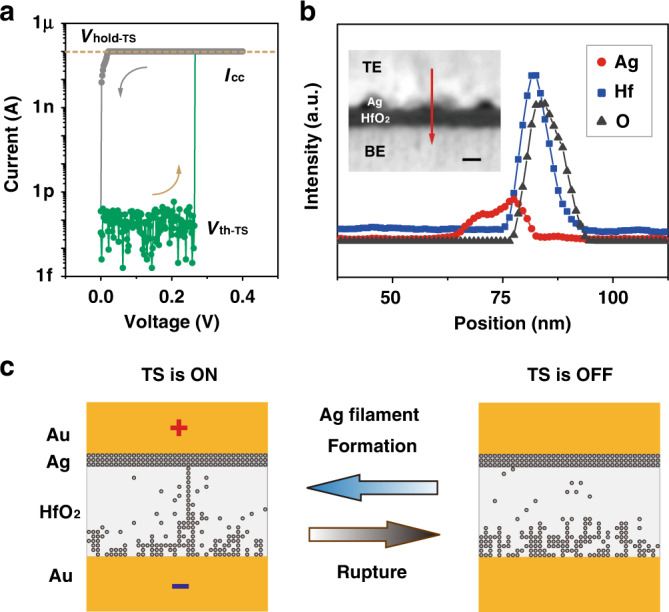
Ag atomic threshold-switching (Ag/HfO_2_-based TS). **a** Typical *I*–*V* characteristic of the TS device at a compliance current (*I*_cc_) of 100 nA in forward (green)/reverse (grey) voltage sweeping, exhibiting ultralow leakage currents (<1 pA). **b** Energy-dispersive X-ray spectroscopy (EDS) line profiles (including Ag, Hf, and O elements) of the TS stack layers along the red line shown in a cross-sectional high-angle annular dark-field scanning transmission electron microscopy (HAADF-STEM) image. Scale bar: 10 nm. **c** Simulation for the formation and rupture procedures (on/off) of the atomic Ag filament in the TS device with an applied voltage of 0.4 V.

The excellent subthreshold characteristics of the ATS-FET are attributed to the series integration of high-performance TS device. Therefore, it is critical to achieving a TS device with superior threshold-switching behavior. The typical *I*–*V* characteristic of the as-fabricated TS device at a compliance current (*I*_cc_) of 100 nA is shown in Fig. 3a. The TS exhibits volatile threshold-switching behavior with a small threshold voltage of the TS (*V*_th-TS_ = ~0.26 V), an ultralow leakage current (<1 pA), and a high on/off current ratio (>10^6^). The TS device switches from the off-state to the on-state at an applied voltage (*V*_a_) larger than *V*_th-TS_ (green curve in Fig. 3a), while it switches to the off-state at *V*_a_ less than the hold voltage (*V*_hold-TS_; grey curve in Fig. 3a). The TS device yields an extremely steep on/off switching slope <0.5 mV decade^−1^, and *V*_th-TS_ ranges between 0.205 and 0.265 V in the cyclic tests. Energy-dispersive X-ray spectroscopy (EDS) line profiles of the cross-sectional TS stack layers are shown in Fig. 3b. The inset is a high-angle annular dark-field scanning transmission electron microscopy (HAADF-STEM) image. The sphere-shaped Ag layer, HfO_2_ dielectric layer, and top/bottom electrode (TE/BE) layers can be clearly observed, with the Ag atomic-scale layer accumulated at the interface of TE/HfO_2_.

To investigate the effect of the filament morphology on the switching dynamics, the formation and rupture of the Ag filament at a *V*_a_ of 0.4 V are illustrated in Fig. 3c via a Monte Carlo simulation (the flow chart is shown in Supplementary Fig. 2). The on-/off-state of the TS is determined by the formation/rupture of Ag filament with volatile threshold-switching behavior. The HfO_2_ electrolyte layer of the TS can be considered as a variable barrier for electron transport. The Ag nanoparticles diffusion into HfO_2_ matrix contributes to electrons transport along the nanoparticle chain (i.e., filament), and the adjacent Ag nanoparticles can act as electron traps for thermionic emission or tunneling^30^. As shown in Supplementary Fig. 3, the Arrhenius curve is used to explain the temperature dependence of the leakage current of a TS device. The charge transport of the TS at HRS is governed by a combination of Frenkel-Poole (F-P) emission and trap-assisted tunneling (TAT) process^31–33^. Specifically, in the high-temperature region (>200 K), the current strongly depends on the temperature, indicating the F-P emission mechanism. In contrast, in the low-temperature region (<200 K), the current shows weak temperature-dependent behavior due to the existence of the TAT mechanism.

By applying a large voltage on the TS, the abrupt formation of the Ag filament commonly induces the NDR effect across the TS. The measured *I*–*V* characteristics of the TS device in voltage-sweeping and current-sweeping modes are shown in Supplementary Fig. 4, indicating good performances for current-controlled (or S-type) NDR behavior. The NDR effect is further illustrated with a control sample (the TS connected with a resistor *R*_L_, Supplementary Fig. 5a) to study the distribution of the voltage drops during the on/off switching process of the TS. The NDR effect induces an abruptly decreased voltage drop (*V*_TS_ − Δ*V*_NDR_) across the TS device, and consequently an amplified voltage drop (*V*_L_ + Δ*V*_NDR_) across the series resistor (Supplementary Fig. 5a). The decrease in the voltage drop (−Δ*V*_NDR_) across the TS device can be extracted from the AC *I*–*V* characteristics (Supplementary Fig. 5b). According to the analysis of the control sample, it can be predicted that the NDR effect can induce a similar internal voltage amplification by replacing the resistor with a MoS_2_ FET.

### Monitoring the internal amplification in the ATS-FET

**Fig. 4:**
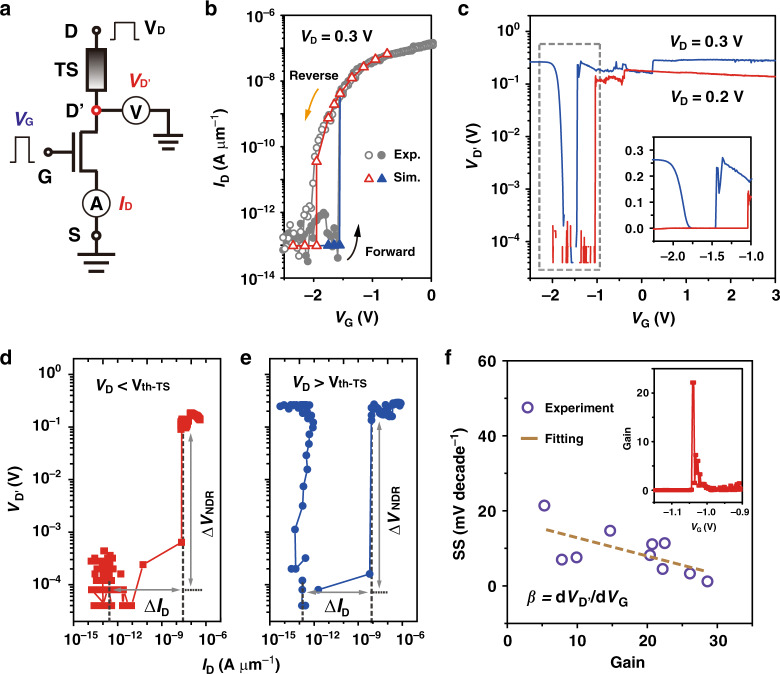
ATS-FET with an internal amplification across the MoS_2_ channel. **a** The equivalent circuit schematic for the electrical measurements. *V*_D′_ and *I*_D_ in red are the output parameters that need to be simultaneously recorded with the increase of the input *V*_G_ (blue). **b** The experimental and simulated transfer (*I*_D_–*V*_G_) characteristics of the ATS-FET at *V*_D_ = 0.3 V, indicating the simulated results are in good agreement with the experimental data. **c** The relations between the gate voltage (*V*_G_) and the internal voltage (*V*_D′_) for two *V*_D_ schemes, including *V*_D_ (= 0.2 V) < *V*_th-TS_ and *V*_D_ (= 0.3 V) > *V*_th-TS_. The inset is the linear scale from the data shown in the dashed grey rectangle. **d**, **e** *V*_D′_ changes as a function of *I*_D_ for the two *V*_D_ schemes. The NDR effect induces an amplification of *V*_D′_ across the MoS_2_ channel. **f** The relation between the average SS and the internal amplification gain (*β* = d*V*_D_/d*V*_G_). The inset indicates the sharp increase in *V*_D′_ with respect to the applied *V*_G_ occurring in the region of the abrupt switching due to the NDR effect.

The equivalent circuit diagram of the ATS-FET can be considered as two variable resistors connected in series, as shown in Fig. 4a. We try to disclose the working mechanism of the ATS-FET by using mathematical derivation and simulation with a semi-quantitative model^34–36^, and the simulation is illustrated in Supplementary Fig. 6 and Note 1. Taking the highly nonlinear *I*–*V* characteristics both of TS and baseline MoS_2_ FET into consideration, the node voltage (*V*_D′_) and the channel current (*I*_D_) can be solved as the intersections of output characteristics (*I*_D_–*V*_D′_, black lines) of the baseline MoS_2_ FET and the *I*–*V* curve (*I*_D_–*V*_D′_, red line) of TS device for different *V*_G_ (see Supplementary Fig. 6c). Impressively, the simulated transfer curves extracted from Supplementary Fig. 6c are in good agreement with the experimental data, as depicted in Fig. 4b. From a physical viewpoint, the steep SS phenomenon is caused by the NDR effect of TS, and it can be understood by a concept of internal amplification gain (*β* = d*V*_D′_/d*V*_G_), which is defined to describe the relationship between *V*_D′_ and *V*_G_. According to the definition of subthreshold swing, SS can be written as

2 $${\mathrm{SS}} 	= \frac{{\partial V_{\mathrm{G}}}}{{\partial {\mathrm{log}}_{10}(I_{\mathrm{D}})}} = \frac{{\partial V_{\mathrm{G}}}}{{\partial V_{{\mathrm{D}{\prime}}}}} \times \frac{{\partial V_{{\mathrm{D}}{\prime}}}}{{\partial {\mathrm{log}}_{10}(I_{\mathrm{D}})}} = \frac{{\frac{{2.3kT}}{q}}}{{\frac{1}{n} + \frac{\beta }{{\exp \left( {\frac{{qV_{{\mathrm{D}}{\prime}}}}{{kT}}} \right) - 1}}}} \\ 	 \approx \frac{{2.3kT}}{q} \times \frac{{\exp \left( {\frac{{qV_{{\mathrm{D}}{\prime}}}}{{kT}}} \right) - 1}}{\beta }\\ 	 \approx 0\,{\mathrm{mV}}\,{\mathrm{decade}}^{ - 1},\,{\mathrm{when}}\,\beta \to \infty ,$$

where *V*_D′_ is the output voltage at the internal node D′, *β* is the internal amplification gain, *n* is the ideal factor, *q* is the basic electron charge, *k* is the Boltzmann constant, and *T* is the absolute temperature. Ideally, *β* is approximated to be infinity (i.e., ∆*V*_G_ = 0) in the forward switching process, and thus SS will be approximated to be zero. However, due to the inevitable limitation by the accuracy of the testing instrument, the measured SS is >0 mV decade^−1^.

To verify and further elaborate the internal amplification induced by the NDR, we monitor the output voltage at the internal node D′ (*V*_D′_) and analyze the voltammetry characteristics under current sweeping at two different *V*_D_. According to *V*_th-TS_ at ~0.26 V, we reasonably select two typical *V*_D_ values (0.2 and 0.3 V, i.e., *V*_D_ < *V*_th-TS_ and *V*_D_ > *V*_th-TS_, respectively). As shown in Fig. 4c, the abrupt increases in *V*_D′_ are clearly observed under both *V*_D_ (*V*_D_ < *V*_th-TS_ and *V*_D_ > *V*_th-TS_). As *V*_G_ sweeps over *V*_T_, the redistribution of the effective potential drops on the MoS_2_ channel and the TS pulls up (increases) *V*_D′_ according to the drain voltage superposition effect. The *V*_D′_ drop on the MoS_2_ channel is amplified according to the NDR effect caused by the TS transition from the off-state to the on-state. Notably, the trends of the variation in *V*_D′_ are obviously distinguishable for different *V*_D_. *V*_D′_ at *V*_D_ = 0.3 V shows an initial decrease with a subsequent increasing trend, while *V*_D′_ at *V*_D_ = 0.2 V only shows an increase at the pull-up point. The pull-up *V*_G_ value also shifts from −1.45 to −1.04 V as *V*_D_ decreases from 0.3 to 0.2 V (inset of Fig. 4c). The underlying reason is explained by the voltammetry characteristics under current sweeping. For *V*_D_ < *V*_th-TS_ (Fig. 4d), the TS is initially in the off-state at *I*_D_ < 1 × 10^−12^ A μm^−1^. To maintain a low current in the series circuit, *V*_D′_ is therefore kept at a low level to minimize the current in the MoS_2_ FET. As *I*_D_ increases, *V*_D’_ slightly increases. When *I*_D_ is large enough (exceeding 1.6 × 10^−12^ A μm^−1^) to trigger the Ag filament formation in the TS, the resistance of the ATS-FET abruptly decreases with *V*_D′_ representing a steep increment (Δ*V*_NDR_). In contrast, *V*_D′_ is initially at a high level (0.263 V) for *V*_D_ > *V*_th-TS_ (Fig. 4e). This may be because the supply voltage applied from the drain electrode is transiently imposed on the TS component and triggers the bridging of the Ag filament. The instantly increased device current results in a high *V*_D′_ at the beginning. Then, *V*_D′_ dramatically decreases with a subsequent slight increasing trend as *I*_D_ sweeps. When *I*_D_ exceeds 1.4 × 10^−12^ A μm^−1^, *V*_D′_ represents a steep increment (Δ*V*_NDR_). As the supply voltage in Fig. 4e is larger than that in Fig. 4d, the trigger current for the Ag filament would be relatively smaller, which is consistent with the results in Fig. 4c.

Furthermore, the internal amplification gain (*β* = d*V*_D′_/d*V*_G_) extracted from the abrupt switching region is ~22.2 for a low supply voltage at *V*_D_ = 0.2 V (the inset of Fig. 4f), corresponding to the SS_min_ of 2.5 mV decade^−1^ and the SS_average_ of 3.0 mV decade^−1^ over four decades of *I*_D_. As the internal amplification gain increases to 28.6, the SS_average_ can be further reduced to a record of 1.3 mV decade^−1^ (Fig. 4f). The internal amplification gain is considered a significant parameter for designing steeper-slope FETs with lower energy consumption.

Based on the above discussion, the internal voltage amplification in the ATS-FET according to the voltage snapback (Δ*V*_NDR_) induced by the NDR effect is directly observed in this work. From the point of view of charge carriers, the free electrons transported in the ATS-FET are blocked by the barrier of the HfO_2_ electrolyte at the beginning (*V*_G_ < *V*_T_). When *V*_G_ reaches *V*_T_, *I*_D_ increases in an abrupt fashion as a result of the Ag filament formation. Hence, the ATS-FET can in principle overcome the fundamental thermionic limitation of 60 mV decade^−1^ at room temperature.

### Improved ATS-FET with much-reduced hysteresis and SS

**Fig. 5:**
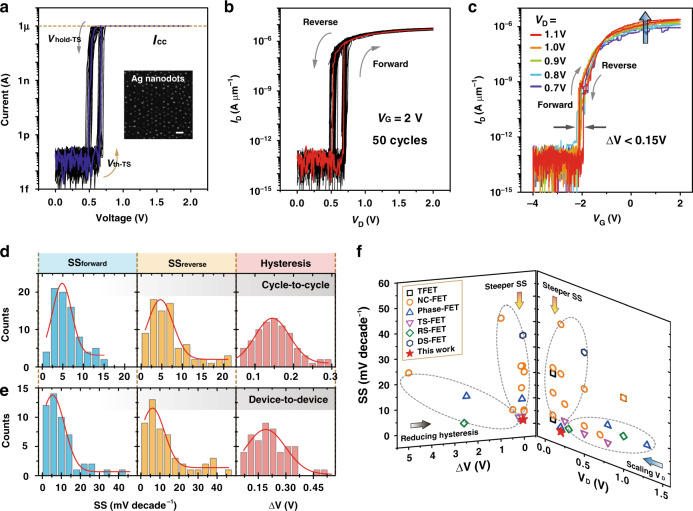
ATS-FET with much-reduced hysteresis and SS. **a** Typical *I*–*V* characteristics of the TS device based on highly ordered Ag nanodots in 30 cycles (*I*_cc_ = 1 μA). The inset is a scanning electron microscopy (SEM) image of highly ordered Ag nanodots. **b** Output characteristics of the ATS-FET with the newly presented TS at a *V*_G_ of 2 V in 50 cycles. 2D MoS_2_ channel (channel width: 2.2 μm; channel length: 1.8 μm). **c** Transfer characteristics (*I*_D_–*V*_G_) of the ATS-FET with the newly presented TS at a *V*_D_ ranging from 0.7 to 1.1 V. **d**, **e** Statistical distributions of SS_forward_, SS_reverse_, and hysteresis (Δ*V*) of the ATS-FET in **d** cycle-to-cycle and **e** device-to-device variations. **f** Comparisons of SS–hysteresis (left) and SS–*V*_D_ (right) in different steep-slope FETs, including the TFET^8,39,40^, NC-FET^18–20,41–46^, phase-FET^21,47,48^, Ag (or Cu) filament TS-FET^22,28,49^, resistive-switching FET (RS-FET)^50,51^, and DS-FET^16^, and our ATS-FET.

Hysteresis is commonly undesirable for transistors in logic applications^37^. Technically, the achievable ATS-FET with small hysteresis (or hysteresis-free) and ultralow subthreshold characteristics is capable to offer the promising potential for ultralow-power logic circuit applications. However, the ATS-FET described above exhibits large hysteresis of 0.5–1 V (Fig. 2), which in essence needs to be greatly reduced. Some device optimization methods were used to reduce the hysteresis, such as annealing, and passivation, as previously reported^37,38^. To suppress the hysteresis of the ATS-FET, we also present an effective approach of device optimization by using the TS device with highly ordered Ag nanodots, which could contribute to shrinking the switching window (e.g., reducing the difference between *V*_th-TS_ and *V*_hold-TS_) and improving *I*_hold-TS_ of TS. The newly presented TS device is fabricated with a HfO_2_ layer of 10 nm, an active electrode of highly ordered Ag nanodots, and followed by a process of rapid thermal annealing. It is critical to treat the atomic Ag layer with rapid thermal annealing to make the Ag atoms accumulate in a spherical shape, which is preferential for interstitial dopants in HfO_2_ to guarantee the volatile threshold-switching behavior even at a high-compliance current^26^. With the compliance currents (*I*_cc_) defined from 10 nA to 50 μA, the TS device shows abrupt and volatile threshold-switching behavior (Supplementary Fig. 7a). And the TS device also exhibits good stability in the cyclic test (Fig. 5a), achieving a reduced switching window of 0.12–0.24 V and small variations both in *V*_th-TS_ and *V*_hold-TS_ (Supplementary Fig. 7b). Furthermore, by connecting such TS device to a MoS_2_ FET, an improved ATS-FET is demonstrated to have much-reduced hysteresis and SS in a highly reproducible manner. The output characteristics (*I*_D_–*V*_D_) of the improved ATS-FET for different *V*_G_ are shown in Supplementary Fig. 8. Fifty continuous cycles of the output characteristics (at *V*_G_ = 2 V) in Fig. 5b can also verify the stable and repeatable operations of the ATS-FET. From the transfer characteristics in Fig. 5c, we can see that the improved ATS-FET shows reduced hysteresis of <0.15 V at different *V*_D_ (ranging from 0.7 to 1.1 V). More impressively, the nearly negligible hysteresis (10 mV) is observed at the *V*_D_ of 0.7 V, meanwhile the SS_min_ in forward and reverse sweeps are 2.6 and 12.5 mV decade^−1^, respectively (Supplementary Fig. 9). Further extracting key parameters, including SS_forward_, SS_reverse_, hysteresis, and *V*_T_, from three ATS-FETs are shown in Supplementary Fig. 10. The SS_forward_, SS_reverse_, and hysteresis are all independent of *V*_D_, while the *V*_T_ shows the negative shift with the increase of *V*_D_ (consistent with that of Fig. 2e).

### Statistical analysis of ATS-FET and the device comparison

To more clearly illustrate the reproducibility of the ATS-FET, we conduct the statistical analysis for the average SS (including SS_forward_ and SS_reverse_), and hysteresis in cycle-to-cycle (80 cycles) and device-to-device (50 devices) variations. Figure 5d shows the histograms and Gaussian fits for the SS_forward_, SS_reverse,_ and hysteresis (4.8 and 4.6 mV decade^−1^; 0.14 V) of the ATS-FET in 80 cycles. Besides, the abrupt switching behavior of the ATS-FET is free of device-to-device deviations. The Gaussian distributions of the SS_forward_, SS_reverse_, and hysteresis are plotted in Fig. 5e, showing that the statistical SS_forward_, SS_reverse,_ and hysteresis mainly distribute at 5.3 mV decade^−1^, 6.1 mV decade^−1^, and 0.19 V, respectively.

According to the above-described Eq. (1), *V*_D_ and SS synergistically contribute to the evaluation of power consumption in the FET device. And hence, it is recommended that both *V*_D_ and SS are preferred to be minimized, in addition to the suppressed hysteresis in transfer curves. Compared with previous reports of different categories of steep-slope transistors, including the TFET^8,39,40^, NC-FET^18–20,41–46^, phase-FET^21,47,48^, Ag (or Cu) filament TS-FET^22,28,49^, resistive-switching FET^50,51^, and DS-FET^16^, the relations of SS–hysteresis and SS–*V*_D_ are both summarized and depicted in Fig. 5f. Capable of achieving steeper SS, reducing hysteresis, and scaling *V*_D_, the ATS-FETs show superior performances by using the atomic-scale geometry design with seamless integration of 2D FET and TS. Specifically, the achieved ATS-FET is nearly hysteresis free (10 mV), and has ultralow SS_min_ of <2.6 mV decade^−1^, which can satisfy the ITRS requirement for the SS of 25 mV decade^−1^ in the year of 2027 (ref. ^1^), and will be promising for future electronics with ultralow-power consuming.

## Discussion

In summary, we successfully demonstrate an ATS-FET that significantly overcomes the fundamental thermionic limitation of the SS. This ATS-FET achieves extremely abrupt steepness in the turn on characteristics with a typical SS_average_ of 4.5 mV decade^−1^ for more than five decades of *I*_D_ at room temperature, and exhibits a significant off-state leakage current reduction to lower the power dissipation. According to the analysis of the *I*–*V* dynamics in the ATS-FET both in experiment and simulation, the NDR effect from the TS transition can contribute to inducing an internal *V*_D′_ amplification across the MoS_2_ channel, enabling the ATS-FET to break the “Boltzmann tyranny”.

As a benefit of the active materials in atomic scale (MoS_2_ and Ag filament), the proposed ATS-FET permits the critical scaling of the supply voltage and exhibits superior electrical performances, as well as greatly suppressed off-state current (Supplementary Fig. 11). In addition, the *V*_D_ can be readily scaled by reducing the HfO_2_ thickness of TS, but the abrupt SS behavior can be maintained independent of the variable HfO_2_ thickness (Supplementary Fig. 12). The geometry design with seamless integration of FET and TS is promising for the potential monolithic integration of the ATS-FET in wafer scale. In this work, the sharp on/off switching properties with ultralow SS in the ATS-FET is originated from the threshold-switching behavior with an internal voltage amplifier, which is universal and applicable to other emerging 2D semiconducting materials related FETs and even different types of transistor devices. To meet the practical applications of logic circuits (also illustrated in the ITRS), the ATS-FETs are available with additional optimization from technical aspects, e.g., further decreasing off current, SS and hysteresis, and improving on/off ratio and reliability. Based on the simulated analysis (Supplementary Fig. 13 and Note 2), the ATS-FET with hysteresis-free behavior is possible to be achieved. Alternatively, in the case of the steep-slope FETs with hysteresis, they could be potentially used as memory devices^52^ or some specific logic circuits, e.g., Schmitt trigger^53^. Looking forward, the achievement of an ultralow-power steep-slope ATS-FET would coordinate with the scientific research of short-channel devices to efficiently reduce power dissipation, which is highly desirable for next-generation energy-efficient integrated circuits and ultralow-power electronics.

## Methods

### Device fabrication

The MoS_2_ nanoflakes were initially synthesized on highly p-doped rigid silicon (Si) substrates with a thermally grown 275 nm-thick SiO_2_ layer through CVD. Copolymer and polymethyl methacrylate was spin-coated at a speed of 4000 r.p.m. and then baked on a hot plate at 150 °C. Subsequently, the source–drain electrodes of the MoS_2_ transistor and the BEs of the TS were simultaneously defined with a standard EBL process, thermal evaporation of Cr/Au (10/40 nm), and lift-off process. A dielectric HfO_2_ layer of 5–20 nm was fabricated via ALD (PICOSUN/SUNALE R-200) approach. A pre-deposited seed layer of 1 nm of Al via thermal evaporation could help to obtain a high-quality dielectric film. The top gate electrodes of the MoS_2_ transistor were defined by a second EBL process and metallization of Cr/Au (10/40 nm). The top electrodes of the TS were defined by a third EBL process and metallization of Ag/Au (3/40 nm).

For the TS device with highly ordered Ag nanodots, an ultrathin AAO template was transferred onto the prepared layers after the step of ALD, and then patterned an Ag thin film (4 nm), and followed by a process of rapid thermal annealing (500 °C for 30 s) after the removal of AAO template. The bottom and top electrodes were fabricated following the above-described process. The as-fabricated TS device can be connected to a baseline FET (MoS_2_, monolayer or multilayer) to construct the ATS-FET.

### Materials characterization and electrical measurements

The Raman spectrum was measured by a HORIBA/LabRAM HR Evolution spectrograph with a 532 nm excitation laser. The cross-sectional HAADF-STEM image and EDS line profiles of the TS were obtained by STEM (FEI Tecnai F20). The SEM image was captured by a FEI Nova 450. And the EBL was performed by the FEI Nova 450 with a Raith pattern generator. The electrical measurements of the transistors were performed on a probe station equipped with a semiconductor parameter analyzer (Agilent B1500A) in an ambient atmosphere at room temperature.

## Supplementary information

Supplementary Information

## Data Availability

All data supporting this study and its findings are available within the article and its Supplementary Information or from the corresponding author upon reasonable request.

## Code availability

The codes that support the findings of this study are available from the corresponding author upon reasonable request.
