# Supplementary material for: Atomic threshold-switching enabled MoS2 transistors towards ultralow-power electronics
Source: Nat Commun. 2020 Dec 4;11:6207. doi: 10.1038/s41467-020-20051-0 (PMC7719160; doi:10.1038/s41467-020-20051-0)
Supplement: Supplementary file 1 — Supplementary Information [file 41467_2020_20051_MOESM1_ESM.pdf]

Supplementary Information

Atomic Threshold-Switching Enabled MoS_2 Transistors towards Ultralow-Power Electronics

Qilin Hua[1,2,3,6], Guoyun Gao[2,3,6], Chunsheng Jiang[1,6], Jinran Yu[2,3], Junlu Sun[2], Taiping Zhang[4], Bin Gao[1]*, Weijun Cheng[1], Renrong Liang[1], He Qian[1], Weiguo Hu[2,3], Qijun Sun[2,3]*, Zhong Lin Wang[2,3,5]*, Huaqiang Wu[1]*

[1] *Institute of Microelectronics, Beijing Innovation Center for Future Chips (ICFC), Tsinghua University, Beijing, 100084, China*

[2] *CAS Center for Excellence in Nanoscience, Beijing Key Laboratory of Micro-nano Energy and Sensor, Beijing Institute of Nanoenergy and Nanosystems, Chinese Academy of Sciences, Beijing, 101400, China*

[3] *School of Nanoscience and Technology, University of Chinese Academy of Sciences, Beijing 100049, P. R. China*

[4] *Department of Electrical Engineering, Tsinghua University, Beijing, 100084, China*

[5] *School of Materials Science and Engineering, Georgia Institute of Technology, Atlanta, GA 30332-0245, USA*

[6] *These authors contributed equally: Qilin Hua, Guoyun Gao, and Chunsheng Jiang.*

* To whom the correspondence should be addressed.
Email: wuhq@tsinghua.edu.cn; sunqijun@binn.cas.cn;
zhong.wang@mse.gatech.edu; gaob1@tsinghua.edu.cn;

Supplementary Table 1 | Performance comparison of different types of steep-slope MoS_2 FETs.

Device	TFET [1]	NC-FET [2]	RS-FET [3]	ATS-FET
Mechanism	Tunnel	Negative capacitance	Resistive switching	NDR
Structure	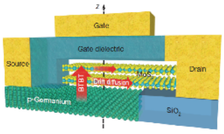	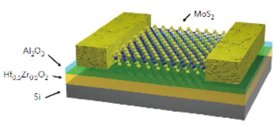	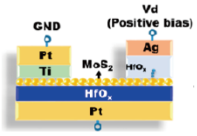	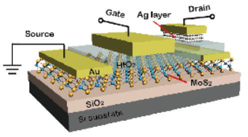
Transfer Curevs	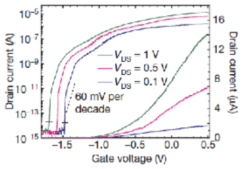	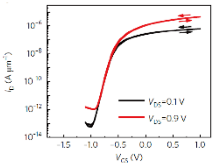	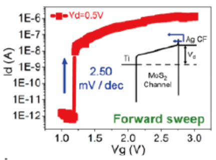	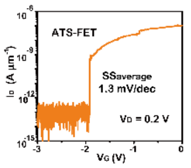
SS vs. I_D	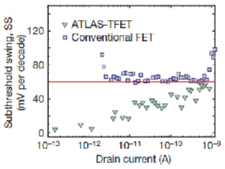	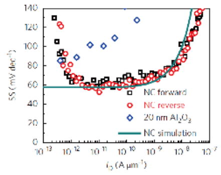		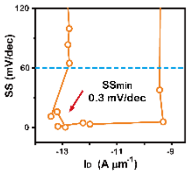
SS_{min}	3.9 mV/dec	$SS_{Forward}$ = 59.6 mV/dec<br>$SS_{Rev,min\#1}$ = 41.7 mV/dec<br>$SS_{Rev,min\#2}$ = 5.6 mV/dec	2.26 mV/dec	0.3 mV/dec
$SS_{average}$	31.1 mV/dec (4 decades)		2.5 mV/dec (4 decades)	1.3 mV/dec (3 decades)

Note: in the case of ATS-FET, the $SS_{average}$ of 1.3 mV decade^{-1} can be achieved, as a result of the internal amplification gain ($\beta = dV_{D'}/dV_G$) increasing to 28.6.

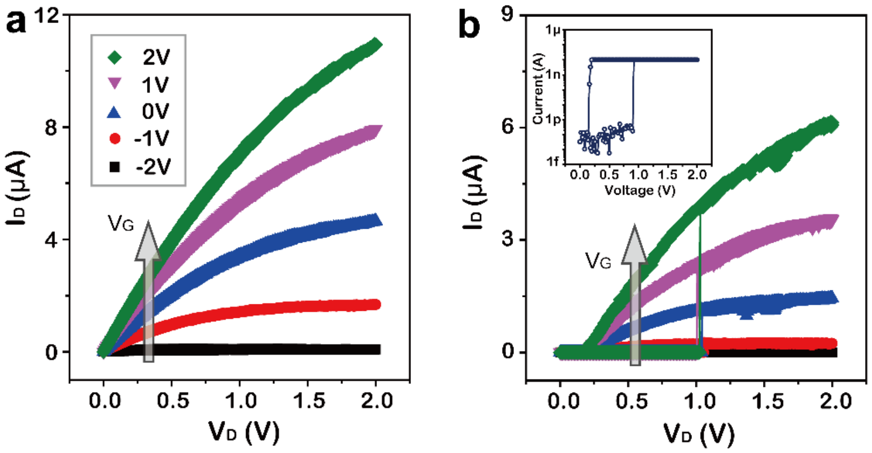

Supplementary Figure 1 | Output (I_D-V_D) characteristics of (**a**) the MoS_2 FET and (**b**) the ATS-FET at different gate voltage V_G (from -2 to 2 V) in linear scale. The inset indicates the *I*-*V* characteristic of the connected TS device, in which the ALD-deposited HfO_2 thickness is 15 nm.

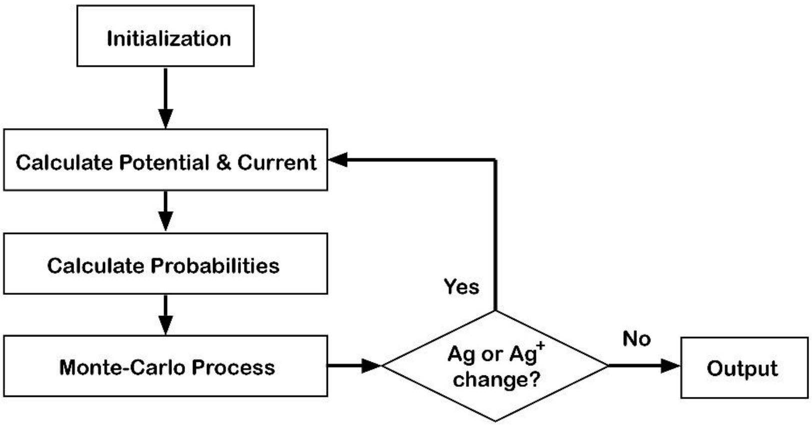

Supplementary Figure 2 | Schematic flow chart of the Monte Carlo simulation for Ag filament formation in the TS device.

Atomic stochastic simulation is introduced to self-consistently investigate the microscopic processes of Ag migration. The distribution of electrical potential and current density can be solved by the resistor network model. The probabilities of generation (oxidation/reduction: $\mathrm{Ag} \rightarrow \mathrm{Ag}^{+} + \mathrm{e}^{-};\ \mathrm{Ag}^{+} + \mathrm{e}^{-} \rightarrow \mathrm{Ag}$) of Ag atoms and migration of Ag^{+} can be given by $P = f \cdot exp(-(E - \Delta\varphi)/k_B T)$, where f is the vibration frequency, E is activation energy of oxidation/reduction or hopping barrier of Ag^{+} respectively, and $\Delta\varphi$ is barrier height reduction induced by electric field.

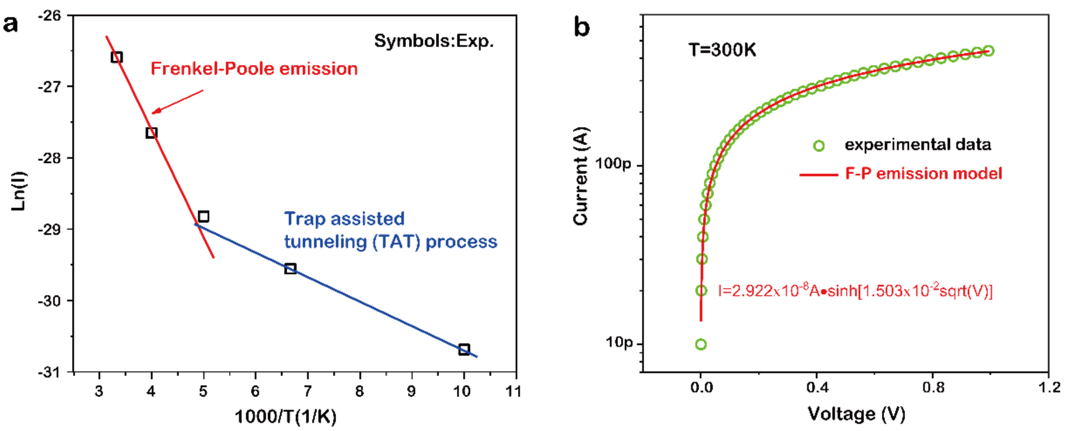

Supplementary Figure 3 | Conduction mechanism of the TS. a, Temperature dependence of leakage current of the TS device. At high temperature (> 200 K), the current strongly depends on the temperature, indicating the Frenkel-Poole (F-P) emission mechanism. At low temperature (< 200 K), the current shows the weak temperature-dependent behavior, indicating the trap assisted tunneling (TAT) mechanism. **b**, Conduction mechanism (at HRS) of the TS device at room temperature can be attributed to the F-P emission.

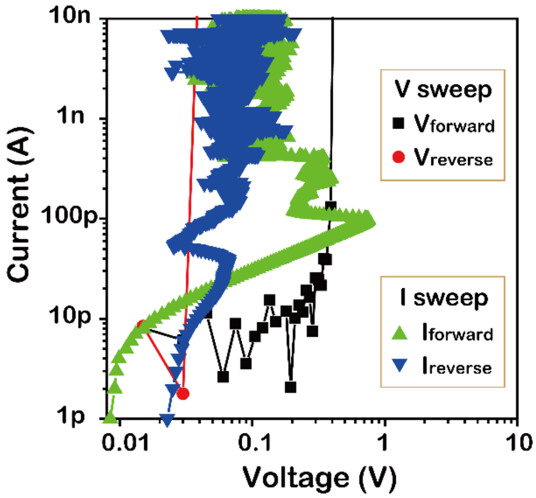

Supplementary Figure 4 | Measured *I-V* characteristics of the TS device in voltage-sweeping and current-sweeping modes. Note that the approximated "S" shape curve cannot be observed under the DC sweep process in voltage-sweeping mode. The current sweep step of 1 pA is determined by the analyzer. Both V_{NDR} in forward/reverse current sweeps are observed with approximated "S" shape, indicating good performances for current-controlled (or S-type) NDR behavior.

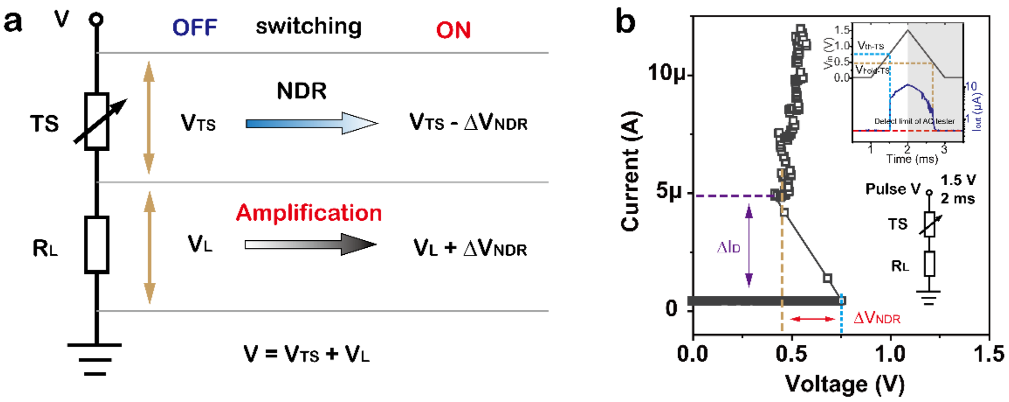

Supplementary Figure 5 | The NDR effect of the TS. a, Circuit schematic of the voltage redistribution between the TS device and a load resistor R_L when the TS switches on or off. V_{TS} and V_L are the voltage drop across TS and R_L, respectively. As the TS switches from the off-state to the on-state, the NDR effect will lead to an abrupt voltage drop (ΔV_{NDR}) across the TS device (= V_{TS} - ΔV_{NDR}) with a consequent amplification across the series resistor R_L (= V_L + ΔV_{NDR}). **b**, The abrupt voltage drop (ΔV_{NDR}) across the TS device is recorded to show the possibility of internal voltage amplification (in forward sweep). The inset shows the measured AC *I-V* characteristics of the TS device connected to a load resistor R_L of 81 kΩ by applying a triangle voltage pulse (1.5 V, 2 ms).

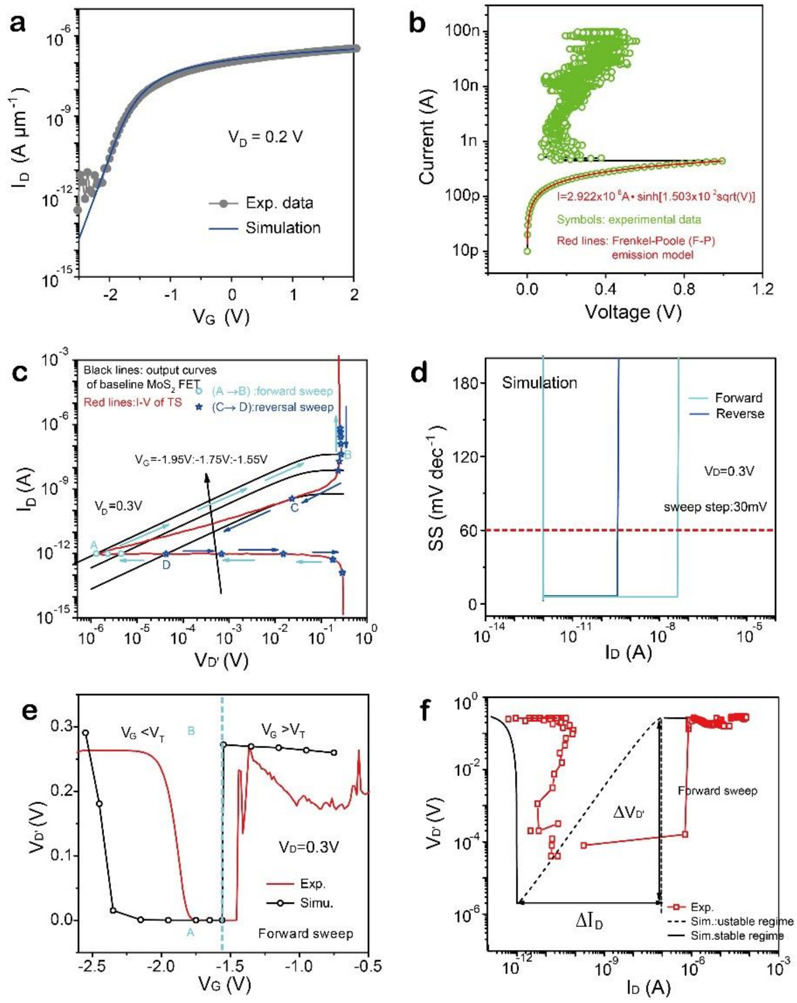

Supplementary Figure 6 | Simulation of the ATS-FET. a, Transfer characteristics of the baseline MoS_2 FET for both the simulation and experiment data when V_D = 0.2 V. Symbols represent experiment measurement and lines represent simulation results. Simulation results make a good agreement with the experiment measurement. **b**, Experimented *I*-*V* characteristics of the TS device in current-sweeping mode. The red fitting curve indicates the conduction mechanism of TS at HRS is in accordance with the Frenkel-Poole (F-P) emission model. **c**, The solution of the voltage ($V_{D'}$) of internal node D' using the graphical method. The node voltage ($V_{D'}$) and current in the channel (I_D) can be seen as the intersections of output characteristics(I_D-$V_{D'}$) of the baseline MoS_2 FET and the *I*-*V* curve (I_D-$V_{D'}$) of TS device. **d**, Extracted *SS*-I_D curves of ATS-FET from c). **e**, The relation between the gate voltage (V_G) and the internal voltage ($V_{D'}$) for both experiment and simulation in the forward sweep process. **f**, The relation between the channel current (I_D) and the internal voltage ($V_{D'}$) for both experiment and simulation in the forward sweep process.

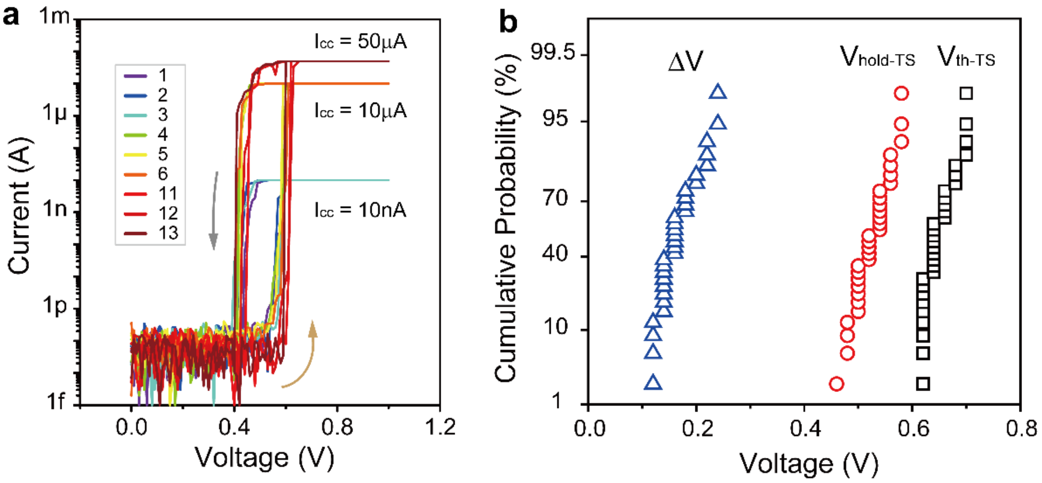

Supplementary Figure 7 | Switching characteristics of the TS device with highly ordered Ag nanodots. a, Continuous cyclic *I-V* characteristics of the TS device at different compliance currents (I_{cc}) from 10 nA to 50 μA, showing abrupt switching behavior from the off-state to the on-state. **b**, Cumulative probability of $V_{th\text{-}TS}$, $V_{hold\text{-}TS}$, and hysteresis (ΔV) of the TS for 30-cycle test, showing the stable switching operations after device optimization.

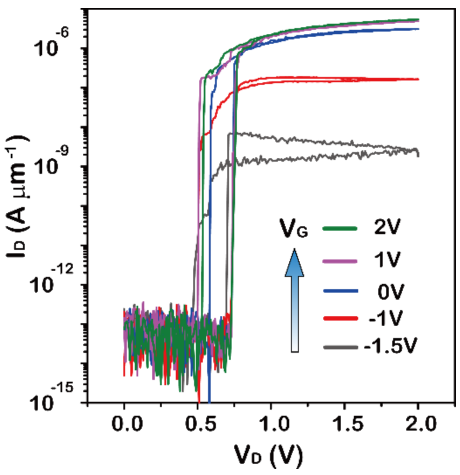

Supplementary Figure 8 | The output characteristics (I_D-V_D) of the improved ATS-FET at different V_G.

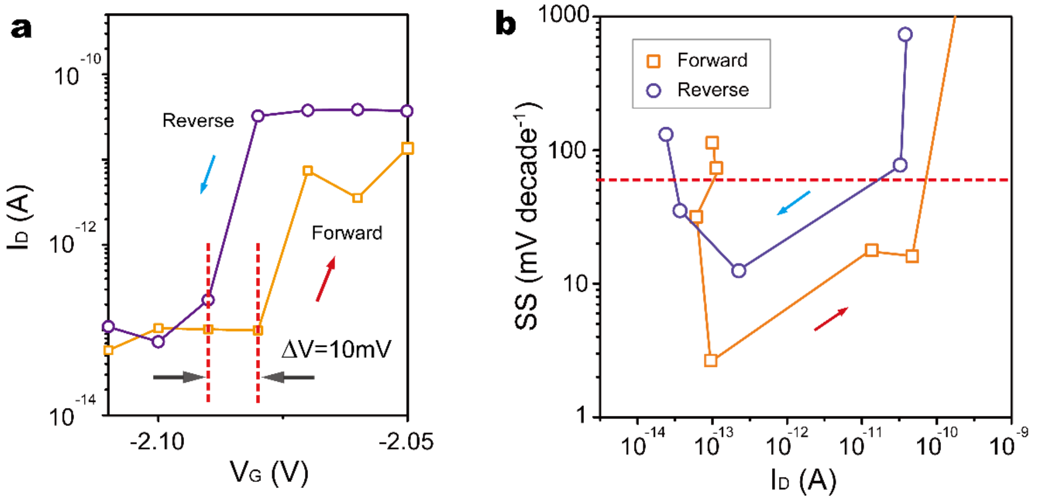

Supplementary Figure 9 | Improved ATS-FET with much-reduced hysteresis and *SS*. **a**, Transfer characteristics (I_D-V_G) of the improved ATS-FET measured at V_D = 0.7 V (also shown in Fig. 5c), indicating a very small hysteresis of 10 mV. **b**, Extracted *SS* curves from the transfer characteristics, demonstrating the SS_{min} in forward and reverse sweeps are 2.6 and 12.5 mV decade^{-1}, respectively.

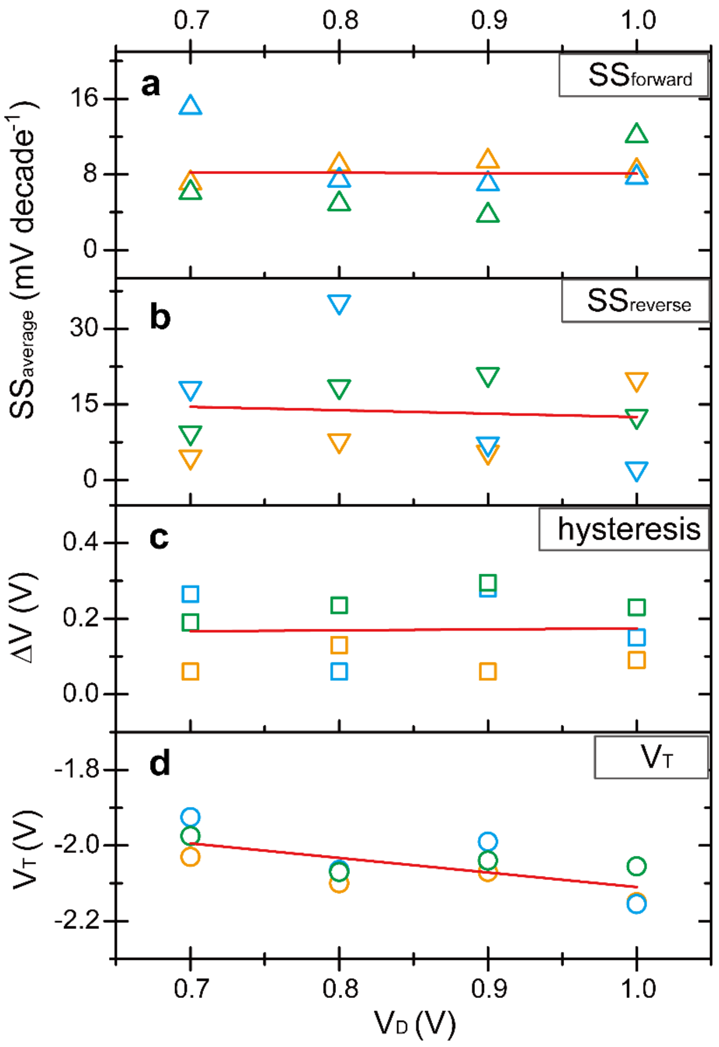

Supplementary Figure 10 | Extracted key parameters from three ATS-FETs (with the TS device based on highly ordered Ag nanodots) at various V_D (from 0.7 to 1 V), including **a**, $SS_{average\_forward}$, **b**, $SS_{average\_reverse}$, **c**, hysteresis, and **d**, V_T.

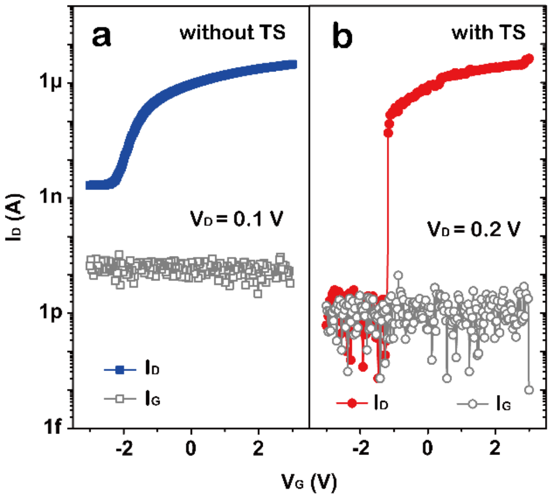

Supplementary Figure 11 | Measured transfer characteristics (in forward sweep) of a 2D MoS_2 FET without (**a**) or with (**b**) the TS connected to the drain side, showing significant suppression of the leakage current (3 orders of magnitude) with the TS configuration (ultrahigh resistance of the TS in the off-state is ~ 1 TΩ).

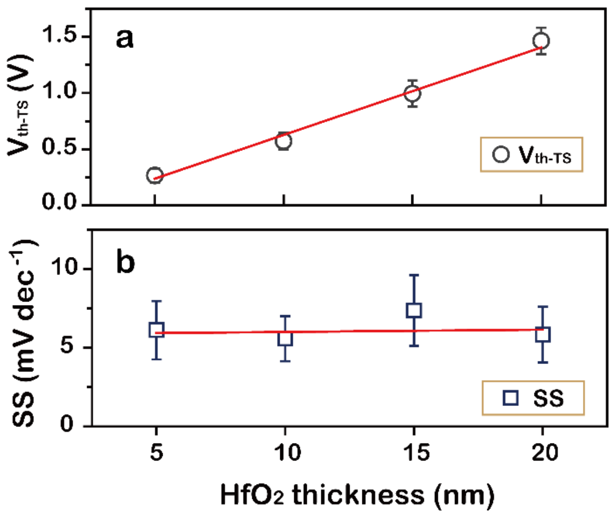

Supplementary Figure 12 | a, The relation between the $V_{th\text{-}TS}$ and the thickness of HfO_2 in the TS device, indicating that the $V_{th\text{-}TS}$ can be tuned by controlling the thickness of HfO_2. **b**, The relation between the average *SS* and the thickness of HfO_2, showing the *SS* is independent of the variable HfO_2 thickness of TS. Error bar indicates five devices used for each test.

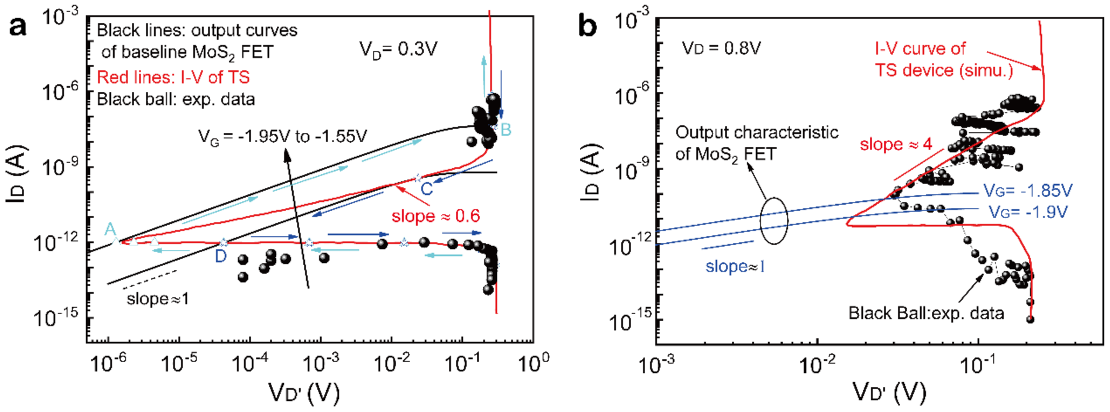

Supplementary Figure 13 | The experiment and simulation relations between the gate voltage (V_G) and the internal voltage ($V_{D'}$) in the forward sweep process for (**a**) Dev. #1 and (**b**) Dev. #2.

Dev. #1 (also shown in Fig. 2): the slope of the NDR regime of TS (~0.6) is smaller than the slope of the output curve (~1) and the numbers of the intersection of two curves can be three, which leads to the hysteresis.

Dev. #2 (also shown in Figs. 5b,c): the slope of the NDR regime of TS (~4) is bigger than the slope of the output curve (~1) and the numbers of the intersection of two curves only can be one, which would lead to the non-hysteresis.

Supplementary Note 1 | Simulation of the ATS-FET

The ATS-FET can be considered as a baseline MoS_2 FET in series with a TS device, as illustrated in Figs. 1b and 4a. From the experimental measurements of the baseline MoS_2 FET, the Schottky barrier heights for source/drain contacts are insignificant at room temperature. The electrical characteristics of the baseline MoS_2 FET can be modeled using the classical drift-diffusion mechanism,[4] while the current-voltage (I-V) curve of the TS device can be modeled based on the theory of metallic filamentary conduction.[5,6] The transfer characteristic of the baseline MoS_2 FET is obtained as Ref. [4]:

$$I_{\mathrm{D}} = q\mu_n \frac{W}{L}\left[c\phi - \frac{d}{2}\phi^2\right]_{\phi_s}^{\phi_d} \tag{1}$$

$$\phi(x) = \frac{b}{a} - v_t W\left\{\frac{1}{av_t} exp\left[\frac{(b-aV_{ch})}{av_t}\right]\right\} \tag{2}$$

$$a = \frac{\epsilon_{2D}T_{2D}}{qDOS\lambda^2} \tag{3}$$

$$b = \left(\frac{\epsilon_{2D}T_{2D}E_0}{q} + N_d\right)/DOS \tag{4}$$

$$c = N_d + \frac{\epsilon_{2D}T_{2D}}{q}(\xi + \frac{kT}{q\lambda^2}) \tag{5}$$

$$d = \frac{\epsilon_{2D}T_{2D}}{q\lambda^2} \tag{6}$$

$$\xi = \frac{(V_{\mathrm{G}} - V_{FB0})}{\lambda^2} \tag{7}$$

$$\lambda = \sqrt{\frac{\epsilon_{2D}t_{ox}T_{2D}}{\epsilon_{ox}}} \tag{8}$$

Where ϕ_d and ϕ_s are calculated from equation (2) for ϕ by setting V_{ch} = 0 V and $V_{D'}$, respectively. q is the basic electron charge, and n_{2D} is the electron density per area in the channel. N_d is the doping concertation in the channel. ϵ_{2D} is the permittivity of MoS_2. T_{2D} is the thickness of MoS_2 channel. m^* is the effective electron mass, k is the Boltzmann constant, and T is the absolute temperature. $\hbar$ is the reduced Planck constant. V_{ch} is the quasi-fermi potential which

is 0 V and $V_{D'}$ at the source and drain terminals, respectively. V_{FB0} is the flat-band voltage. $W(x)$ is the lambert function, which satisfies the equation $W(x)exp[W(x)]=x$. μ_n is the effective electron mobility. v_t is the thermal voltage, defined as kT/q. DOS is a temperature-dependence constant, defined as $\frac{m^*kT}{2\pi\hbar^2}$. Model parameters are extracted by the fitting method from our experiment as shown in Supplementary Figure 6a.

The TS shows an I-V curve with an approximated "S" shape, indicating the negative differential resistance (NDR) effect, in the experiment under the DC sweep process in the current-mode, as shown in Supplementary Figure 6b. Note that the approximated "S" shape curve cannot be directly observed under the DC sweep process in voltage-mode and the classical hysteresis can be seen in this case. Exact modeling of the I-V curve of TS is very difficult due to the stochastic nature of ions diffusion. In this work, the I-V curve of the TS device is constructed based on the theory of metallic filamentary conduction with some critical data observed in the experiments.[5,6] According to this theory, the high resistance state (HRS) can be modeled properly considering the Frenkel-Poole (F-P) emission model (Supplementary Figure 6b). We try to disclose the working principle of the ATS-FET using this semi-quantitative model.

As shown in Supplementary Figure 6c, both taking the highly nonlinear I-V characteristics of the TS device and the baseline MoS_2 FET into consideration, the node voltage ($V_{D'}$) and the channel current (I_D) can be solved as the intersections of output characteristics (I_D-$V_{D'}$, black lines) of the baseline MoS_2 FET and the I-V curve (I_D-$V_{D'}$, red line) of TS device for different gate voltage (V_G). Moreover, as shown in Fig. 4b, the transfer characteristics of ATS-FET can be extracted from Supplementary Figure 6c. It can be seen that the simulated results are in good

agreement with the experimental data.

Two important conclusions must be highlighted.

(1) The origin of hysteresis for the ATS-FET

As shown in Supplementary Figure 6c, there exists only one intersection of two *I-V* curves when V_G is smaller than -1.95 V or bigger than -1.55 V, which leads to a monotonous I_D-V_G curve. However, there exist three intersections of two *I-V* curves when V_G is between -1.95 V and -1.55 V. In addition, only two of three intersections are stable under DC voltage sweep,[7,8] which leads to a non-monotonic I_D-V_G curve (*i.e.*, hysteresis phenomenon).

(2) The origin of zero subthreshold swing

Theoretically, the subthreshold swing of the ATS-FET would be zero in ideal situation for both forward and reverse sweeps. When the baseline MoS_2 FET works in the subthreshold regime (the ATS-FET also works in the subthreshold regime in this case), the drain current described by equation (1) can be reduced to [9]

$$I_\mathrm{D} = \mu_n \frac{W}{L} v_t \sqrt{q N_d \pi v_t 2\epsilon_{2D}} \exp\left[\frac{(V_\mathrm{G}-V_\mathrm{G0})}{n v_t}\right] \cdot \left[1 - \exp\left(-\frac{V_{\mathrm{D}'}}{v_t}\right)\right] \tag{9}$$

$$= \mathrm{I}_0 \exp\left[\frac{V_\mathrm{G}}{n v_t}\right] \cdot \left[1 - \exp\left(-\frac{V_{\mathrm{D}'}}{v_t}\right)\right]$$

$$\mathrm{n} = \left(1 + \frac{C_{it}}{C_{ox}}\right) = \left(1 + \frac{q^2 D_{it}}{C_{ox}}\right) \tag{10}$$

Herein, v_t is the thermal voltage, defined as *kT/q*. *n* is called the ideal factor, which represents the impact of the interfacial trap density (D_{it}) and *n* is estimated as 2~3 from the experiments. In the forward sweep, $V_{D'}$ has to be reduced when V_G increases to keep a relatively small off-state current, because the TS device works at the HRS, as shown in Supplementary Figure 6e.

When V_G increases to -1.55 V, the working state of ATS-FET jumps from *A* to *B with a constant* V_G while $V_{D'}$ and I_D go up obviously. One can see that the increment of I_D is only caused by the increment of $V_{D'}$ in this process as shown in equation (9). The subthreshold swing (*SS*) can be defined as

$$SS_{\text{ideal}}(\text{A} \rightarrow \text{B}) = \frac{\Delta V_G}{\Delta log_{10}(I_D)} = \frac{0\text{V}}{\Delta log_{10}(I_D)} = 0 \ \text{mV/dec} \tag{11}$$

However, the actual DC measurement is a dynamic process because the sweep step of V_G cannot be zero ($\Delta V_G \neq 0V$). As a result, the measured value of *SS* is slightly larger than 0 mV decade^{-1} dependent on the particular sweep step of V_G, as shown in Supplementary Figure 6d. Similarly, the reverse sweep process ($C \rightarrow D$) can be analyzed as well.

Note that this steep subthreshold swing (slope) is caused by the NDR effect of the TS device from a physical viewpoint. Because in the forward switching process ($A \rightarrow B$), the conduction current (I_D) of TS increases abruptly while the voltage drop on the TS device ($V_\text{D} - V_{\text{D}'}$) decrease abruptly, which is a typical NDR effect. This steep subthreshold swing (slope) can also be understood by the concept of the internal amplification gain ($\beta = \text{d}V_{D'}/\text{d}V_G$). According to the definition of subthreshold swing, *SS* can be rewritten as

$$SS_{\text{Baseline-FET}} = \frac{\partial V_G}{\partial log10(I_D)} = \frac{2.3nkT}{q} \tag{12}$$

$$\begin{aligned} SS_{\text{ATS-FET}} &= \frac{\partial V_G}{\partial log_{10}(I_D)} = \frac{\partial V_G}{\partial V_{D'}} \cdot \frac{\partial V_{D'}}{\partial log_{10}(I_D)} = \frac{1}{\beta} \cdot \frac{\partial V_{D'}}{\partial log_{10}\left\{\text{I}_0 \exp\left[\frac{V_\text{G}}{nv_t}\right] \cdot \left[1 - \exp\left(-\frac{V_{\text{D}'}}{v_t}\right)\right]\right\}} \\ &= \frac{1}{\beta} \cdot \frac{2.3kT}{q} \cdot \frac{1}{\frac{1}{n\beta} + \frac{1}{\exp\left(\frac{qV_{D'}}{kT}\right)-1}} = \frac{2.3kT}{q} \cdot \frac{1}{\frac{1}{n} + \frac{\beta}{\exp\left(\frac{qV_{D'}}{kT}\right)-1}} \approx \frac{2.3kT}{q} \cdot \frac{\exp\left(\frac{qV_{D'}}{kT}\right)-1}{\beta} \\ &\approx 0 \ \text{mV/dec} \ \text{when} \ \beta \rightarrow \infty \end{aligned} \tag{13}$$

As shown in Supplementary Figure 6d and 6e, β is approximated to be infinity (e.g. $\Delta V_\text{G} =$

0) in the forward switching process ($A \rightarrow B$). As a result, *SS* is approximated to be zero. Thus, the internal amplification gain (β = d$V_{D'}$/dV_G) is also a result of the NDR effect of TS device.

In practical terms, $SS_{ATS\text{-}FET}$ is smaller than 2.3nkT/q (~$SS_{Baseline\text{-}FET}$), which is induced by $V_{D'}$ modulated by V_G, meanwhile the abrupt increase of I_D is closely/directly related with the large increase of $V_{D'}$ (instead of V_G) in the switching process.

Supplementary Note 2 | Analysis of hysteresis-free in the ATS-FET

The ATS-FET has the possibility to realize the hysteresis-free operation. As stated in ref. [10], the hysteresis-free operation can be achieved by matching the resistance of the MOSFET (R_S) and the negative differential resistance (R_{TS}) of TS device. Once the condition of $R_S > |R_{TS}|$ is satisfied, the hysteresis-free operation can be achieved in the ATS-FET.

To explain the physical mechanism of hysteresis-free operation more clearly, we define a similar parameter, S (slope), which is defined as $\partial log_{10}(I_{\text{sub}})/\partial log_{10}(V_{D'})$ in the log-log coordinates as shown in Supplementary Figure 13. Two different ATS-FETs have been fabricated, denoted as Dev. #1 and Dev. #2. For the baseline MoS_2 FET, the S parameter can be obtained from its output characteristics. In the subthreshold regime, the subthreshold current (I_{sub}) can be expressed as

$$I_{\text{sub}} = \mathrm{I}_0 \exp\left[\frac{V_{GS}}{nv_{th}}\right] \cdot \left[1 - \exp\left(-\frac{V_{D'}}{v_{th}}\right)\right] \tag{14}$$

where I_0 is a constant and dependent on the specific geometric dimension and fabrication process of the baseline MoS_2 FET, v_{th} is called as thermal voltage ($= \frac{kT}{q}$). In the case of $V_{D'} < v_{th}$, the Eq. (14) is simplified to

$$I_{\mathrm{sub}} \approx \mathrm{I}_0 \exp\left[\frac{V_{GS}}{nv_{th}}\right] \cdot \frac{V_{D'}}{v_{th}} \leftrightarrow S = \partial log_{10}(I_{\mathrm{sub}})/\partial log_{10}(V_{D'}) \approx 1 \quad (15)$$

It can be observed that the S (slope) of the output curve of the MoS_2 FET is about 1 for a given gate voltage for a small $V_{D'}$ in the log-log coordinates as shown in Supplementary Figure 13. For Dev. #1 as shown in Supplementary Figure 13a, the S of the NDR regime of TS device (~0.6) is smaller than the slope of the output curve (~1) and the numbers of the intersection of two curves could be three, which leads to the hysteresis. However, for Dev. #2 as shown in Supplementary Figure 13b, the S of the NDR regime of TS (~4) is bigger than the slope of the output curve (~1) and the numbers of the intersection of two curves only can be one, which would lead to the hysteresis-free operation.

Note that the slope of the NDR regime in the TS device is dependent on its threshold voltage ($V_{th\text{-}TS}$), threshold current ($I_{th\text{-}TS}$), hold voltage ($V_{hold\text{-}TS}$), and hold current ($I_{hold\text{-}TS}$), which can be tuned by optimizing its geometric dimension and fabrication process.[11-13]

References

1 Sarkar, D. *et al.* A subthermionic tunnel field-effect transistor with an atomically thin channel. *Nature* **526**, 91-95 (2015).

2 Si, M. *et al.* Steep-slope hysteresis-free negative capacitance MoS_2 transistors. *Nat. Nanotechnol.* **13**, 24-28 (2018).

3 Wang, X.-F. *et al.* Two-mode MoS_2 filament transistor with extremely low subthreshold swing and record high on/off ratio. *ACS Nano* **13**, 2205-2212 (2019).

4 Jiang, C. *et al*. A closed form analytical model of back-gated 2-D semiconductor negative capacitance field effect transistors. *IEEE J. Electron Devi.* **6**, 189-194 (2017).

5 Wang, W. *et al*. Volatile resistive switching memory based on Ag ion drift/diffusion—Part II: Compact modeling. *IEEE T. Electron Dev.* **66**, 3802-3808 (2019).

6 Wang, W. *et al*. Modeling of switching speed and retention time in volatile resistive switching memory by ionic drift and diffusion. *2019 IEEE Int. Electron Devices Meet.* https://dx.doi.org/10.1109/IEDM19573.2019.8993625 (IEEE, 2019).

7 Jimenez D, Miranda E, Godoy A. Analytic model for the surface potential and drain current in negative capacitance field-effect transistors. *IEEE T. Electron Dev*. **57**, 2405-2409 (2010).

8 Jiang, C. *et al*. A carrier-based analytical theory for negative capacitance symmetric double-gate field effect transistors and its simulation verification. *J. Phys. D Appl. Phys.* **48**, 365103 (2015).

9 Duarte, J. P., Choi, S. J., Choi, Y. K. A full-range drain current model for double-gate junctionless transistors. *IEEE T. Electron Dev.* **58**, 4219-4225 (2011).

10 Shukla, N. et al. A steep-slope transistor based on abrupt electronic phase transition. *Nat. Commun.* **6**, 7812 (2015).

11 Wang, Z. *et al.* Threshold switching of Ag or Cu in dielectrics: materials, mechanism, and applications. *Adv. Funct. Mater.* **28**, 1704862 (2018).

12 Midya, R. *et al.* Anatomy of Ag/Hafnia-based selectors with 10^{10} nonlinearity. *Adv. Mater.* **29**, 1604457 (2017).

13 Hua, Q. *et al.* A threshold switching selector based on highly ordered Ag nanodots for X-point memory applications. *Adv. Sci.* **6**, 1900024 (2019).
